# The impacts of the COVID-19 pandemic on adults who experience imprisonment in Greece – A qualitative study

**DOI:** 10.1192/j.eurpsy.2023.496

**Published:** 2023-07-19

**Authors:** L. Tsitsoni, K. Argyropoulos, D. Avramidis, G. Charalambous, P. Gourzis, E. Jelastopulu

**Affiliations:** ^1^Postgraduate Programme “Health Management, University of Frederick, Nicosia, Cyprus; ^2^Public Health, School of Medicine, University of Patras, Greece; ^3^School of Medicine, Univesrity of Patras, Patras, Greece; ^4^Psychiatry; ^5^Public Health, School of Medicine, Univesrity of Patras, Patras, Greece

## Abstract

**Introduction:**

The prison environment and health of people who experience incarceration increase the risks of contracting COVID-19. Aside from the risks of infection and transmission, one should also examine the impact on their mental health state and identify their needs, since prison inmates experience already disadvantages and inequalities to a large extent.

**Objectives:**

The aim of the study was to explore the experiences of inmates with the pandemic and the restrictions, their insights into the pandemic and the impact of social distancing in prison on their emotional status and management.

**Methods:**

We used in-depth interviews with 5 inmates and 6 prison employees of a state prison in Athens, to produce an analysis of the challenges that the inmates faced during the pandemic. The study took part from February till June 2022. Each participant was asked semi-structured questions and a thematic content analysis was performed.

**Results:**

The results of the interviews revealed several key themes, that have emerged from the COVID-19 pandemic. The challenges that the participants faced were in relation to communication, feelings of heightened isolation and detachment from family, friends, and the normal rhythms of life in and out of prison. Furthermore, our study has shown that COVID-19 pandemic resulted in higher levels of anxiety, lack of positive stimuli, of work and education, of day-to-day interaction, and of information.

**Conclusions:**

This study highlights the dramatic reduction in opportunities for prisoners to rehabilitate themselves and build productive and meaningful lives. A decline in the emotional, psychological and physical well-being of the prisoners as well as resignation with their situation can be expected due to the lack in purpose. There is a need to improve the information and communication and support them with continuous psychological care, especially when prison services are confronted with additional health or other crises.

**Disclosure of Interest:**

None Declared

